# One-year outcomes in patients undergoing very high-power short-duration ablation for atrial fibrillation

**DOI:** 10.1007/s10840-023-01520-0

**Published:** 2023-03-10

**Authors:** Francesco Solimene, Teresa Strisciuglio, Vincenzo Schillaci, Alberto Arestia, Gergana Shopova, Armando Salito, Giuseppe Bottaro, Giovanni Marano, Fernando Coltorti, Giuseppe Stabile

**Affiliations:** 1 Clinica Montevergine, Mercogliano, AV Italy; 2 Department of Advanced Biomedical Sciences, University of Naples Federico II, Naples, NA Italy; 3 Mediterranea Cardiocentro, Naples, NA Italy; 4 Clinica San Michele, Maddaloni, CE Italy; 5 Anthea Hospital, Bari, BA Italy

**Keywords:** Atrial fibrillation, Pulmonary vein isolation, Very high-power short-duration, Catheter ablation, Radiofrequency ablation

## Abstract

**Background:**

The very high-power short-duration (vHPSD) temperature-controlled ablation (vHPSD) improves the efficiency of pulmonary vein isolation (PVI) procedures.

We evaluated the procedural and 12-months outcomes in atrial fibrillation (AF) patients undergoing PVI by means of vHPSD ablation. In patients with AF or atrial tachyarrythmia (AT) recurrence undergoing a redo procedure the durability of the PVI was investigated.

**Methods:**

Consecutive paroxysmal/persistent AF patients undergoing PVI with the vHPSD ablation strategy (90 W, for 4 s) were enrolled. The rate of PVI, first-pass isolation, acute reconnection, and procedural complications were evaluated. Follow-up examinations and EKG were scheduled at 3,6, and 12 months. In case of AF/AT recurrence, patients underwent a redo procedure.

**Results:**

Overall, 163 AF patients (29 persistent and 134 paroxysmal) were enrolled. The PVI was reached in 100% of patients (88% at the first pass). The rate of acute reconnection was 2%. The radiofrequency, fluoroscopy and procedural times were respectively 5.5 ± 1 min, 9 ± 1 min and 75 ± 20 min. No death, tamponade nor steam pops occurred; however, 5 patients had vascular complications. The 12-months freedom from AF/AT recurrence was 86% in both paroxysmal and persistent patients. Overall, 9 patients underwent a redo procedure, and in 4 all veins were still isolated, whereas in 5 pulmonary vein reconnections were found. The PVI durability was 78%. No overt clinical complications were observed in the follow-up.

**Conclusions:**

The vHPSD ablation represents an effective and safe ablation strategy to achieve PVI. The 12-months follow-up showed high freedom from AF/AT recurrence and a good safety profile.

## Introduction

Radiofrequency (RF) energy is the widest energy source used for catheter ablation of arrhythmias [1]. The very high-power short-duration (vHPSD) catheter is a novel contact force (CF) catheter optimized for temperature-controlled ablation with microelectrodes and 6 thermocouples for real-time temperature monitoring during ablation. The associated vHPSD algorithm modulates power to maintain target temperature during lesion formation (maximum temperature 65 °C by using 90 W for 4 s) [2]. A vHPSD strategy of RF ablation aims to minimize conductive heating and increase resistive heating in order to deliver targeted heating to the atrial wall, achieving uniform and transmural lesion while reducing the risk of collateral tissue damages [3, 4]. The vHPSD ablation produces much less conductive heat leading to wider, shallower lesion formation, and much less reversible injury. So far, preliminary data on the novel vHPSD ablation mode showed promising results and demonstrated its safety and efficacy for pulmonary vein isolation (PVI) in patients undergoing atrial fibrillation (AF) ablation [5–8]. However, these studies included only a limited number of patients and long follow-up data are missing. The aim of our study is to evaluate the efficacy and safety of the vHPSD ablation on a larger cohort of patients and to investigate the 12-months outcomes of this strategy.

## Methods

### Study population

This prospective study enrolled consecutive AF patients undergoing their first pulmonary vein ablation between September 2020 and December 2021. The study protocol conforms to the ethical guidelines of the 1975 Declaration of Helsinki and was approved by the Institutional Ethics Committees. Participants included patients who were ≥ 18 years old at the time of presentation with drug-resistant AF, regardless of its duration. Each patient provided informed consent to participate in the study.

The exclusion criteria were: inability to provide informed consent, AF potentially attributable to a cardiac or noncardiac reversible cause (dyselectrolytaemia, dysthyroidism, severe anaemia, sepsis, acute cardiac ischemia and others), previous AF ablation, patients waiting for heart transplant or other cardiac surgery, previous cardiac surgery in the last 3 months, known atrial myxoma, unstable angina, unstable heart failure, life expectancy < 12 months, pregnancy and breastfeeding.

### Study protocol

#### Pulmonary veins ablation

All procedures were performed under general anaesthesia or under deep sedation. Vitamin K antagonist treatment (with a target INR of 2.0–3.0 on the day of the procedure) or non-vitamin K anticoagulants were uninterrupted.

Through femoral venous access, a diagnostic catheter was placed in the coronary sinus (CS) and was used as a timing reference for the creation of high-density electroanatomic maps. After a single transseptal puncture, an open-irrigated tip catheter (the QDOT Micro™ catheter, Biosense Webster, Inc, CA, USA) was inserted into the LA through the hole created, and the fixed-curve sheath (SL0, Abbott) was used to insert the mapping catheter (Pentaray, Biosense Webster, Inc, CA, USA). The left atrial geometry was reconstructed using a 3D electro-anatomic mapping system (Carto Biosense Webster, USA) during pacing from the CS. In patients in AF, electrical cardioversion was performed to restore sinus rhythm before starting the acquisition of the map.

The encirclement of the veins was performed in a point-by-point fashion, by delivering RF energy (90 Watts for 4 s) when a contact force (CF) ≥ 5 g was reached. The target interlesion distance was ≤ 4 mm at the anterior wall, in order to have overlapping of the tags, whereas at the posterior wall was ≤ 6 mm [7]. This strategy aimed to create deeper lesions on the anterior wall that is thicker. The intervenous ablation line was performed at the discretion of the operator.

**Fig. 1:**
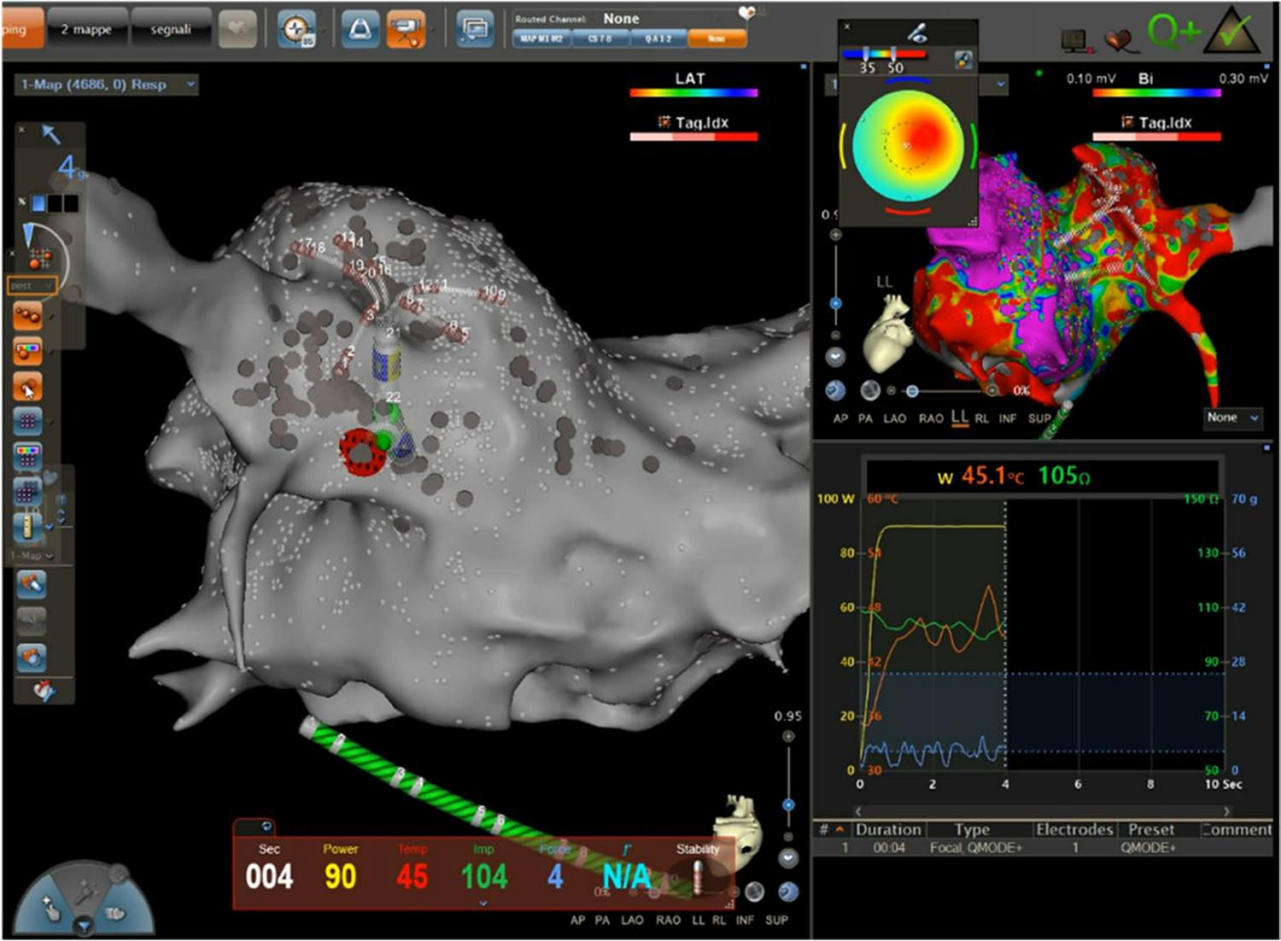
Radiofrequency delivery with very high-power short duration (90 W for 4 s). The left panel shows a tilted postero-anterior view of the left atrium with the ablation catheter delivering the RF energy posteriorly at the ostium of the left inferior pulmonary vein and the Pentaray catheter at the ostium of the left superior pulmonary vein. The right panel shows superiorly a latero-lateral view of the left atrium with the bipolar voltage map, and inferiorly there is a graph showing the temperature curve (in yellow), the impedance curve (in green) and the contact force (in blue) during the ablation

The QDOT Micro™ catheter has an improved irrigation system that includes backward flow toward the proximal electrode, allowing increased irrigation during ablation in a parallel orientation. Ablation has been performed using a RF generator capable of delivering power up to 100 W with a rapid ramp-up time of ≤ 0.5 s that also provides real-time temperature feedback every 33 ms (nGEN RF Generator, Biosense Webster). The vHPSD algorithm rapidly cycles power based on the hottest surface thermocouple (temperature target at 60 °C, cutoff at 65 °C). During the mapping, the flow is low at 2 ml/min. When a RF application starts, a 2-s pre-RF delay begins for the high flow rate (8 ml/s) to cool the surface tissue prior to the onset of RF delivery. After the 2-s delay, the energy quickly ramps up to 90 W and is automatically terminated after 4 s (Fig. 1). At the end of each application, the high flow continues for 4 s. The energy of 90W is delivered for the entire 4 s if the target temperature is not reached. If, during the 4-s application, the target temperature is reached, the power is modulated down to prevent overheating. The power continues to be modulated in response to the temperature measured for the duration of the application. If the temperature cut-off is reached, the RF application is stopped immediately.

Once the encirclement of the vein was completed the PVI was assessed by entrance block and by remapping with the Pentaray catheter.

In case the PVs were not isolated at first pass, the earliest signal on the mapping catheter was targeted for ablation until the PVI was reached. After a waiting time of 20 min, the PVI was reassessed and in case of reconnection touch-up, RF applications were delivered, until re-isolation. The cavo-tricuspid isthmus (CTI) line was performed only in case the patient had a documented typical atrial flutter.

#### Post-ablation management and follow-up

An interrupted oral anticoagulation strategy was pursued in all patients. After ablation, the oral anticoagulation was administered for at least 3 months and long life in patients with a CHA2DS2-VASC score ≥ 2. Patients with paroxysmal AF were discharged without antiarrhythmic drugs. Patients with persistent AF were discharged with or without antiarrhythmic drugs according to the clinician’s preference. Patients were scheduled for clinical and ECG follow-up examinations at 3,6, and 12 months, and then every 6 months, after the initial treatment. A Holter monitoring was performed at 3, 6, and 12 months and in case of symptoms as palpitations. In case the patients were unable to come to the visits, trans-telephonic interviews were performed.

An AF/AT recurrence was considered when documented by an ECG or when clinical symptoms potentially related to AF/AT were reported by the patient. As early relapse within the first 3 months after RF ablation may be a transient phenomenon, this transition period was excluded from the final analysis.

A redo procedure was scheduled in case of AF/AT recurrence out of the blanking period. During \the redo procedure a careful remap was performed in order to depict PV reconnections. In case all the veins were still isolated a more antral ablation was performed or extra PV ablations were targeted, depending on the operators’ choice. In case of PV reconnections, the ablations were delivered at the sites of earliest signals until complete PVI.

#### Study endpoints

The primary outcomes we evaluated were (1) the rate of “First Pass” isolation and the rate of complete PVI at the end of the procedure, (2) the safety of the procedure, (3) the 12-months outcomes including freedom from AF/AT recurrence and the occurrence of any complication, and (4) the PVI durability in redo procedures.

The secondary outcomes we evaluated included overall procedural duration (defined as the time from the first venous puncture to the withdrawal of the sheath), radiofrequency time and fluoroscopy time.

#### Statistics

Continuous variables are expressed as mean ± standard deviation or median and interquartile range according to their distribution. Normality of data distribution was tested with the Shapiro–Wilk test. Categorical variables are expressed as absolute number with percentage (%). The rate of freedom from any atrial tachyarrhythmias was assessed by using the Kaplan–Meier curve. Statistical significance was set at a 2-tailed probability level of < 0.05. All statistical analyses were performed using SPSS software (Version 24.0, IBM, Armonk, NY, US).

## Results

### Population

Overall, 163 patients were enrolled in the study (29 persistent AF, 134 paroxysmal AF). Clinical characteristics are shown in Table 1. The cohort included 105 men and 58 women with a mean age of 61 ± 8 and a CHA_2_DVAS_2_ score of 2 ± 1.

**Table 1:** Clinical characteristics of the study population

	*N* = 163
Age (years)	61 ± 8
Male sex (%)	105 (64%)
AF type	
○ Paroxysmal ○ Persistent	134 (82) 29 (18)
Dyslipidemia (%)	24 (15)
Hypertension (%)	98 (60)
Diabetes mellitus (%)	10 (6)
Obesity (%)	5 (3)
Ischemic heart disease (%)	4 (3)
TIA/stroke (%)	1 (1%)
CHA_2-_DS_2-_VASc score (mean)	2 ± 1
LA diameter, mm	43 ± 10
Left common ostium (%)	24 (15%)

*TIA*, transient ischemic attack; *LA*, left atrium

### Procedural data

The procedures were performed under general anaesthesia in all patients except for 5 that received deep sedation. With a median number of 80 RF tags (IQR 74–90), the PVI could be reached in all patients and in 88% of cases at the first pass. The median impedance drop was 10.8 (IQR 8.5–13) Ohms, the median contact force was 9.2 (IQR 5.9–14) g, and the median maximum temperature was 47 (IQR 44–50.3) °C. In 27 patients an additional CTI was performed for a documented typical atrial flutter. In 2% of patients, acute PV reconnections (one at anterior left superior PV, one at anterior left inferior PV, one at posterior right inferior PV) were observed and the earliest signal on the Pentaray catheter was targeted for ablation until complete signal disappearance. The procedural time (skin to skin) was 75 ± 20 min, the RF time was 5.5 ± 1 min and the fluoroscopy time was 9 ± 6 min. No steam pops, tamponade, death nor stroke occurred, however 5 patients experienced an access site-related vascular complication.

### Follow-up

**Fig. 2:**
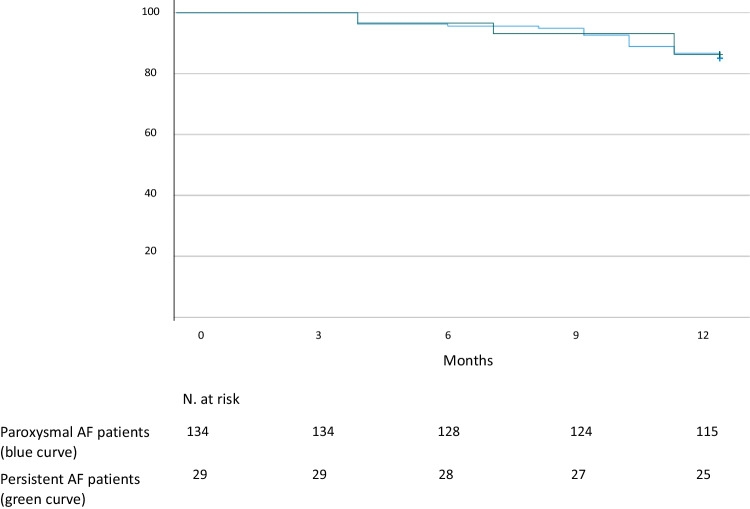
Kaplan–Meier curves showing 12-months freedom from AF/AT recurrence in paroxysmal and persistent AF patients

All patients completed the 12-months follow-up. After a mean follow-up of 19 ± 2 months the freedom from any atrial tachyarrhythmias recurrence was 86% in both cohorts of paroxysmal (18 AF recurrence and 1 patient had electrocardiographic evidence of both AF and atrial flutter recurrence) and persistent AF patients (3 AF recurrence and 1 atrial flutter /tachycardia) (Fig. 2). The mean time to recurrence was 14 ± 4 months. No overt clinical complications were reported during the follow-up.

### Redo procedures

Nine patients underwent a redo procedure for AF/AT recurrence. In 4 patients all the veins were still isolated, whereas in the remaining 5 patients, one reconnected vein was observed in 4 patients and 2 reconnected veins in 1 patient. Thus, 28/36 veins were still isolated and the PVI durability was 78%. The sites of reconnection are described in Table 2.

**Table 2:** Site of PV reconnections at redo procedures

Patient	Reconnected PV				Sites of reconnection			
	RSPV	RIPV	LSPV	LIPV	RSPV	RIPV	LSPV	LIPV
1	0	0	0	0	0	0	0	0
2	0	0	1	0	0	0	Ridge; Anterior roof	0
3	0	0	0	0	0	0	0	0
4	1	0	1	0	Anterior	0	Ridge	0
5	0	0	0	0	0	0	0	0
6	0	0	0	0	0	0	0	0
7	1	0	1	0	Posterior roof	0	Ridge	0
8	0	0	1	0	0	0	Anterior roof	0
9	1	0	1	0	Anterior	0	Ridge	0

*PV*, pulmonary vein; *RSPV*, right superior pulmonary vein; *RIPV*, right inferior pulmonary vein; *LSPV*, left superior pulmonary vein; *LIPV*, left inferior pulmonary vein

## Discussion

This study demonstrated that the use of the vHPSD ablation (90W, 4 s) for PVI is effective and increases the efficiency with short procedural and RF times, without compromising procedural safety. Furthermore, in our cohort of patients, the freedom from AF/AT recurrence at 12 months was high and also the safety profile was confirmed by the absence of overt complications during the follow-up. The PVI durability evaluated at redo procedures was 83%.

### Efficacy data

In recent years, higher-power RF delivery has been proposed as a strategy to improve the efficiency of PVI procedures, by shortening their duration [9]. The point-by-point ablation, indeed, may be time-consuming, and increasing the power enables to reach faster the target ablation index. However, the safety window is narrower, especially for the risk of collateral damage to the oesophagus, when ablating the posterior wall. Indeed, some data on the use of 45/50 W reported the occurrence of steam pops and also oesophageal lesions detected by endoscopy [9, 10].

The QDOT catheter is optimized for temperature-controlled radiofrequency ablation, as it has embedded at the tip microelectrodes and 6 thermocouples that enable a real-time monitoring of the temperature at the tip-tissue interface during the ablation. In the QMODE + modality, the new catheter delivers RF energy with 90 Watts in 4 s (vHPSD), and during the ablation the power is modulated based on the temperature, and this reduces the risk of overheating [11]. Another advantage is that the RF application is so short that catheter stability may not be a concern anymore.

Data on the use of vHPSD for PVI are still limited; however, they confirm the increased procedural efficiency with reduced procedural, fluoroscopy and RF times [5–8]. Our results confirm the shortened procedural times compared to the ablation index-guided procedures [5].

As for the acute procedural success, a low rate of first-pass isolation has been reported by previous studies [6–8] and this has raised concerns also for the durability of the PVI. The study by Nakagawa et al. [12] reported that the applications delivered with the QDOT result in smaller lesion dimensions compared to conventional 50 W-10 s or 30 W-30 s applications; however, this study also suggested that there is a sort of thermal latency, indeed after RF termination the tissue temperature increases due to a slower conductive heating, and thus the lesion size is influenced by the neighbouring RF application and this may create deeper lesions. For this reason, we always aim to create overlapping lesions (interlesion distance ≤ 4 mm) at the anterior wall of the left atrium, which is thicker compared to the posterior wall, and this may account for the high rate of first pass isolation that we report in this study, which is comparable to that reported for point-by-point AI-guided ablation in the multicentric VISTAX study [13]. Furthermore, the general anaesthesia that we systematically perform almost in every patient, increases the catheter stability during the RF delivery thus increasing the probability to reach an effective lesion.

### Twelve-months freedom from AF/AT recurrence and PVI durability

Our data also provide insights into the long-term outcomes of the vHPSD ablation, which seems to be highly effective with 84% of paroxysmal AF patients free from AT/AF recurrence, in line with previous studies reporting on HPSD [10]. As for the PVI durability, in our cohort of patients undergoing a redo procedure, 78% of PVs were still isolated, with most of the reconnections occurring at the superior veins. These data raise enthusiasm on the long-term effectiveness of the vHPSD ablation strategy, also because the rate of durable PVI is much higher than the previously reported with moderate power moderate duration (MPMD) [14]. A similar result was reported also by Yavin et al., that reported a lower incidence of chronic PV reconnection at redo procedures for the HPSD compared to the MPMD (16.6% vs. 52.2%) [15].

### Safety data

The good safety profile previously described for the QDOT catheter [5–8] has been confirmed by our data. No deaths, cardiac tamponade, perforation, stroke, or atrio-oesophageal fistula were observed acutely and during the 12 months follow-up. Previous studies reported the occurrence of silent cerebral events [6, 8] detected by cerebral MRI; however, we did not investigate this aspect.

### Limitations

The study has the following several limitations: (1) this is a prospective mono-centric non-randomized study; (2) the number of enrolled patients is relatively small; however, to our knowledge, there are no other data regarding the vHPSD with 90W for 4 s for PVI in larger cohorts; (3) the adenosine/isoproterenol challenge was not performed at the end of the PVI, and thus, the dormant PV conduction has not been excluded; (4) the impact of the vHPSD on the oesophagus and the brain was not investigated with a oesophagogastroduodenoscopy nor a cerebral MRI; however, in the follow-up, none of the patients had symptoms suggestive of oesophageal nor cerebral injury; (5) no intra-cardiac monitors were used for the assessment of the AF recurrences, thus, the absence of a continuous rhythm monitoring may have underestimated the rate of recurrences.

## Conclusion

The vHPSD ablation represents an effective and safe ablation strategy to achieve PVI. The 12-months follow-up showed high freedom from AF/AT recurrence and a good safety profile with the absence of overt clinical complications.

## Data Availability

Data are available upon reasonable request.
